# Supplementary material for: Parameters of Off-Vertical Axis Rotation in Unilateral and Bilateral Vestibulopathy and Their Correlation with Vestibular Evoked Myogenic Potentials
Source: J Clin Med. 2021 Feb 13;10(4):756. doi: 10.3390/jcm10040756 (PMC7917591; doi:10.3390/jcm10040756)
Supplement: Supplementary file 1 [file jcm-10-00756-s001.pdf]

Table S1. Bias and modulation components on CW and CCW directions in 10 normal subjects.

Patients	Bias (°/s)			Modulation (°)	
	**CW**	**CCW**	**| CW | + | CCW |**	**CW**	**CCW**
No.1 (F/21)	−4.69	3.16	7.85	2.10	3.18
No.2 (F/27)	−4.12	0.47	4.59	2.47	2.60
No.3 (M/47)	−5.93	9.38	15.31	5.42	4.62
No.4 (M/25)	−4.22	3.22	7.44	3.63	3.18
No.5 (F/26)	−1.44	1.54	2.98	3.31	2.69
No.6 (M/24)	−0.21	2.68	2.89	2.77	2.49
No.7 (F/23)	−2.80	2.30	5.1	5.19	5.35
No.8 (M/25)	−3.34	3.14	6.48	0.82	1.39
No.9 (M/41)	−2.05	2.44	4.49	4.63	5.25
No.10 (F/60)	−2.20	0.91	3.11	2.12	2.89
**Mean**	**−3.10**	**2.92**	**6.02**	**3.25**	**3.36**
**SD**	**1.70**	**2.46**	**3.72**	**1.49**	**1.29**

CW, clockwise rotation; CCW, counter-clockwise rotation; SD, standard deviation.

Table S2. Bias and modulation components on CW and CCW directions in 20 right UVH patients.

Patients		Bias (°/s)			Modulation (°)	
	UW(%)	CW	CCW	| CW | + | CCW |	CW	CCW
**No.1 (F/71)**	100	**1.3**	0.9	2.2	**1.88**	1.54
No.2 (F/33)	100	**1.74**	1.63	3.37	**1.15**	1.62
No.3 (M/46)	100	**1.89**	1.65	3.54	**1.59**	1.92
No.4 (M/56)	100	**1.32**	1.08	2.4	**3.43**	4.22
No.5 (F/56)	99	**−0.62**	3.11	3.73	**3.74**	3.70
No.6 (F/65)	83	**−1.03**	−0.2	1.23	**4.39**	4.91
No.7 (M/45)	75	**1.81**	1.16	2.97	**3.14**	2.39
No.8 (F/72)	100	**−0.11**	−0.06	0.17	**4.39**	3.70
No.9 (F/61)	71	**−0.8**	0.1	0.9	**8.17**	8.32
No.10 (F/59)	88	**−0.32**	3.25	3.57	**3.69**	3.94
No.11 (F/42)	100	**−0.01**	3.16	3.17	**3.97**	2.93
No.12 (M/45)	100	**2.53**	2.26	4.79	**9.39**	12.89
No.13 (F/52)	27	**−0.82**	1.01	1.83	**4.7**	3.01
No.14 (F/45)	100	**−0.32**	0.85	1.17	**6.07**	2.1
No.15 (F/68)	79	**−0.88**	0.02	0.9	**6.81**	5.25
No.16 (F/30)	100	**1.83**	2.94	4.77	**2.85**	3.07
No.17 (F/62)	81	**0.69**	2.33	3.02	**3.94**	4.25
No.18 (F/62)	96	**−0.99**	0.47	1.46	**1.76**	3.46
No.19 (F/46)	93	**3.33**	4.96	8.29	**5.25**	5.01
No.20 (M/55)	93	**0.14**	2.39	2.53	**2.78**	1.65
**Mean**	89.25	**0.53**	1.65	2.80	**4.15**	3.99
**SD**	17.52	**1.33**	1.38	1.82	**2.15**	2.64

UW, unilateral weakness (%) in caloric test; CW, clockwise rotation; CCW, counter-clockwise rotation; SD, standard deviation. *Values with bold character are regarded as the affected side bias components.

Table S3. Bias and modulation components on CW and CCW directions in 21 left UVH patients.

Patients		Bias (°/s)			Modulation (°)	
	UW (%)	CW	CCW	∣CW∣+∣CCW∣	CW	CCW
No.21 (M/36)	100	−2	**−0.49**	2.49	2.02	**1.79**
No.22 (M/45)	100	−1.47	**−0.58**	2.05	2.88	**2.16**
No.23 (F/24)	87	−6.21	**−3.01**	9.22	1.00	**0.68**
No.24 (M/36)	100	−1.29	**−0.06**	1.35	2.32	**3.23**
No.25 (F/44)	87	−2.70	**−0.2**	2.9	2.46	**2.39**
No.26 (M/69)	100	−2.33	**−1.96**	4.29	3.34	**3.54**
No.27 (F/35)	98	−3.77	**−1.1**	4.87	6.95	**5.46**
No.28 (F/60)	77	−1.65	**−2.32**	3.97	4.06	**3.28**
No.29 (M/50)	100	−1.27	**−2.53**	3.8	1.01	**2.99**
No.30 (F/56)	84	−1.47	**−0.86**	2.33	3.29	**0.65**
No.31 (F/55)	99	−2.97	**−2.69**	5.66	5.07	**4.47**
No.32 (M/59)	98	0.63	**0.37**	1	4.02	**4.99**
No.33 (M/56)	69	−4.68	**−2.78**	7.46	12.91	**7.54**
No.34 (F/55)	95	−2.50	**−3.04**	5.54	4.66	**7.48**
No.35 (F/42)	68	0.67	**2.84**	3.51	5.07	**4.69**
No.36 (F/33)	97	−2.29	**−2.07**	4.36	2.82	**2.61**
No.37 (M/54)	100	−3.37	**−2.29**	5.66	3.18	**2.89**
No.38 (F/56)	58	−1.72	**0.01**	1.73	1.58	**0.32**
No.39 (M/56)	86	−3.74	**−0.79**	4.53	5.38	**2.64**
No.40 (M/32)	78	−1.70	**1.43**	3.13	1.53	**1.27**
No.41 (F/35)	93	−8.51	**−8.63**	17.14	3.56	**3.71**
Mean	89.24	−2.59	**−1.46**	4.62	3.77	**3.28**
SD	12.67	2.07	**2.25**	3.50	2.60	**1.99**

UW, unilateral weakness (%) in caloric test; CW, clockwise rotation; CCW, counter-clockwise rotation; SD, standard deviation. *Values with bold character are regarded as the affected side bias components.

Table S4. Bias and modulation components on CW and CCW directions in 13 BVP patients.

Patients	Caloric test, Sum of SPV (warm+cold) (°/s)		Bias (°/s)			Modulation (°)	
	Rt	Lt	CW	CCW	∣CW∣+∣CCW∣	CW	CCW
No.1 (M/76)	0	0	−0.53	−0.36	0.89	3.57	1.25
No.2 (F/50)	1	1	−0.24	0.29	0.53	3.03	0.64
No.3 (M/76)	0	0	1.97	0.35	2.32	1.46	0.26
No.4 (F/56)	2	2	0.10	0.19	0.29	4.25	3.95
No.5 (M/35)	0	0	−0.24	0.17	0.41	3.94	1.46
No.6 (F/29)	0	0	−0.24	0.33	0.57	4.62	1.78
No.7 (F/41)	0	2	1.11	1.12	2.23	4.69	2.72
No.8 (M/50)	0	−1	-0.45	0.15	0.6	1.64	2.14
No.9 (M/52)	6	5	−0.37	1.15	1.52	2.80	2.21
No.10 (M/39)	7	0	−1.00	−1.75	2.75	3.00	2.28
No.11 (M/52)	0	7	−0.82	−0.62	1.44	2.30	3.03
No.12 (F/33)	9	9	0.83	0.11	0.94	2.24	1.96
No.13 (F/38)	10	9	−1.48	−0.70	2.18	5.67	5.02
Mean	2.69	2.92	−0.10	0.03	1.3	3.32	2.21
SD	3.84	3.60	0.93	0.77	0.85	1.27	1.29

CW, clockwise; CCW, counter-clockwise; SD, standard deviation.

Table S5. Bias component of OVAR and amplitude of cVEMP and oVEMP in 13 BVP patients .

Patients	Bias (°/s)		N-amplitude of cVEMP		n10–p15 amplitude of oVEMP	
	CW	CCW	Rt	Lt	Rt	Lt
No.1 (M/76)	−0.53	(−0.36)	1.4	1.1	0	0
No.2 (F/50)	−0.24	0.29	1.8	2.2	0	0
No.3 (M/76)	(1.97)	0.35	1.4	1.1	0	0
No.4 (F/56)	0.10	0.19	0	0	0	0
No.5 (M/35)	−0.24	0.17	0	0	0	0
No.6 (F/29)	−0.24	0.33	0	0	0	0
No.7 (F/41)	1.11	1.12	0	0	0	0
No.8 (M/50)	-0.45	0.15	0	0	22	0
No.9 (M/52)	−0.37	1.15	0.8	1.2	28	50
No.10 (M/39)	−1.00	(−1.75)	0	0	0	0
No.11 (M/52)	−0.82	(−0.62)	3.3	2.6	17	14
No.12 (F/33)	(0.83)	0.11	3.7	4.2	35	39
No.13 (F/38)	−1.48	(−0.70)	1.0	4.0	21	34

CW, clockwise; CCW, counter-clockwise. *Figures in parenthesis represent results showing opposite sign.
